# Weaker land–atmosphere coupling in global storm-resolving simulation

**DOI:** 10.1073/pnas.2314265121

**Published:** 2024-03-12

**Authors:** Junhong Lee, Cathy Hohenegger

**Affiliations:** ^a^Max Planck Institute for Meteorology, Hamburg 20146, Germany

**Keywords:** land–atmosphere coupling, soil moisture–precipitation feedback, storm-resolving model, explicit convection

## Abstract

The debate on the sign of the soil moisture–precipitation feedback remains open. On the one hand, studies using global coarse-resolution climate models have found strong positive feedback. However, such models cannot represent convection explicitly. On the other hand, studies using km-scale regional climate models and explicit convection have reported negative feedback. Yet, the large-scale circulation is prescribed in such models. This study revisits the soil moisture–precipitation feedback using global, coupled simulations conducted for 1 y with explicit convection and compares the results to coarse-resolution simulations with parameterized convection. We find significant differences in a majority of points with feedback that is weaker and dominantly negative with explicit convection. The model with explicit convection is more often in a wet regime and prefers the triggering of convection over dry soil in the presence of soil moisture heterogeneity, in contrast to the coarse-resolution model. Further analysis indicates that the feedback not only between soil moisture and evapotranspiration but also between evapotranspiration and precipitation is weaker, in better agreement with observations. Our findings suggest that coarse-resolution models may not be well suited to study aspects of climate change over land such as changes in droughts and heatwaves.

## Significance

The soil moisture–precipitation feedback plays an important role in shaping climate patterns over land. However, the sign of this feedback remains a topic of ongoing debate. We revisit the soil moisture–precipitation feedback using global, coupled simulations conducted at a grid spacing of 5 km. By avoiding the limitations associated with regional models or convective parameterization, our approach offers unique insights. The feedback in the 5-km simulation appears weaker and more negative compared to the one estimated from global coarse-resolution climate models. One potential implication of our findings is that the coarse-resolution climate model, with their strong positive feedback, may overestimate changes in droughts and heatwaves under future climate scenarios.

Already at the turning of the 20th century, Aughey (1) and others (2–4) started to wonder whether soil moisture affects precipitation. In principle, the link between soil moisture and precipitation seems straightforward: more soil moisture leads to more evapotranspiration, more moisture in the atmosphere, and more precipitation. However, it turned out that the direct moistening effect of soil moisture is negligible over many regions of Earth, as most water vapor that feeds precipitation over a region stems from advected moisture and not from locally evaporated moisture (5, 6). Still, indirectly, soil moisture can affect the amount of precipitation, first by affecting the propensity of convection to develop (7–13). Depending on the morning stratification of the atmosphere, either warming (dry soil) or moistening (wet soil) promotes the development of convection. Second, the presence of heterogeneous soil moisture conditions can generate thermally direct, shallow circulations, from the wet to the dry soil. These circulations trigger convection and precipitation on the dry soil (14–17). Thus, it is not obvious whether an increase in soil moisture leads to an increase in precipitation, and how strong this response may be. This may be masked by other processes that control the development of precipitation.

Past studies with coarse-resolution climate models, which have to rely on a convective parameterization to represent convection due to grid spacings coarser than 10 km, have reported a preference for wet soils (18–22). As precipitation itself replenishes soil moisture, this maintains a positive feedback between soil moisture and precipitation. Consistent with the existence of such intrinsic positive feedback, further studies have highlighted the link between spring soil moisture deficit and summer precipitation deficit (23, 24), the amplification of heat wave and temperature variability by soil moisture (20, 24, 25), or the strong decline of precipitation following a reduction in evapotranspiration due to land surface changes such as Amazon deforestation (26). These similar results have been obtained despite the use of different coarse-resolution climate models, integrated in different configurations, but all of these models had to employ a convective parameterization. Yet, convection is at the heart of the soil moisture–precipitation feedback.

Hohenegger et al. (27) were the first to report, using limited-area simulations, that the soil moisture–precipitation feedback depends upon the treatment of convection: negative in simulations with explicit convection and positive in simulations with parameterized convection. Their convective parameterization led precipitation to be sensitive to surface moistening rather than to surface heating. Follow-up studies confirmed the dependency of the feedback sign on the treatment of convection by using regional climate simulations over the Alps (28) and superparameterizations (29). Furthermore, focusing on the feedback between soil moisture heterogeneity and convection, Taylor et al. (30) found negative feedback in observations and positive feedback in coarse-resolution global climate simulations. Large-eddy simulations (31–33) and km-scale models with explicit convection (34, 35) can well reproduce the triggering of convection by such soil moisture heterogeneities. By looking at the partitioning of tropical precipitation between land and ocean, Hohenegger and Stevens (36) deduced a negative feedback between water storage and precipitation in observations and a positive feedback in the ensemble mean of coarse-resolution global climate models used for the Coupled Model Intercomparison Project phase 6 (CMIP6).

Although there is increasing evidence that coarse-resolution climate models may misrepresent the soil moisture–precipitation feedback, studies that have looked at the feedback in simulations with explicit convection were constrained by the limited size of the simulations’ domains. However, interactions between the atmosphere and the land surface vary with the scale of the soil moisture anomaly, large-scale circulation, and oceanic forcing (28, 37–39). Here, we take advantage of global, coupled simulations conducted for 1 y with explicit convection, at a grid spacing of 5 km, with the ICON model (40), to look at land–atmosphere interactions anew. We focus on the sign and strength of the soil moisture–precipitation feedback, quantified by its local coupling via correlation coefficient, during the boreal summer. As documented in two previous studies (40, 41), this storm-resolving version of ICON reproduces well the latitudinal variations of precipitation and its seasonal migration over land. We compare these results with a 60-y simulation conducted with ICON using a coarse grid spacing of 160 km (42), which is representative of the typical behavior of coarse-resolution global climate models as it employed parameterized convection. The hydroclimate of the two models, in terms of time series of soil moisture, precipitation, and evapotranspiration for specific domains, is shown in *SI Appendix*, Figs. S1 and S2 for reference.

## Results

### Weaker and More Negative Soil Moisture–Precipitation Feedback.

**Fig. 1.**
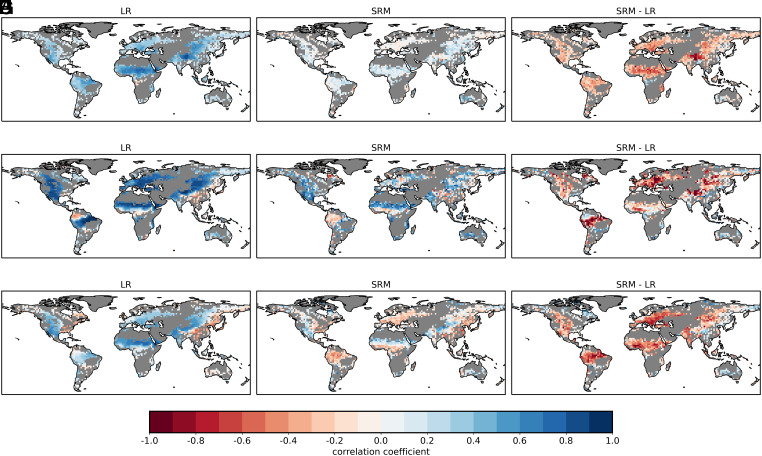
Correlation coefficients computed based on daily mean values for June–July–August between (*A*–*C*) SMI and precipitation, (*D*–*F*) SMI and evapotranspiration, and (*G*–*I*) evapotranspiration and precipitation for the 60-y mean of the coarse-resolution model (LR), the SRM, and the resulting difference in correlation coefficient (*Materials and Methods*). Areas where precipitation is smaller than 0.1 mm d^−1^ in both simulations (called non-rainy area, corresponding to 21.8% of the land area) or where the SMI-precipitation correlation coefficient of the SRM is within one SD of the year-to-year variability of the correlation coefficient in the coarse-resolution model (53.2% of the rainy area) are masked in gray.

Fig. 1*A*–*C* shows the correlation coefficient between soil moisture index (SMI) and precipitation, taken as a measure for the feedback strength (*Materials and Methods*). As we only have 1 y of simulation at storm-resolving resolution (5 km), we use the SD of the 60 correlation coefficients for each individual year of the coarse-resolution model as a measure of internal variability and to assess the significance of the difference between the two models. We exclude the results in cases where precipitation is smaller than 0.1 mm d^−1^ in both simulations or where the SMI-precipitation correlation coefficient of the storm-resolving model (SRM) falls within one SD of the year-to-year variability of the correlation coefficient of the coarse-resolution model. Given this definition, 46.8% of the rainy area exhibits a difference in feedback between the two models. For our subsequent analysis, we only consider those points and will always give percentage values with respect to this subset of points except if noted otherwise.

Fig. 1*A* clearly indicates that the soil moisture–precipitation feedback is weaker in the SRM than in the coarse-resolution model: 90.1% of the points display a weaker correlation. Moreover, 78.2% of the points in the SRM exhibit a correlation coefficient close to zero, with values comprised between ±0.2, whereas a similar fraction of points (70.1%) exhibit a correlation coefficient larger than 0.2 in the coarse-resolution model. As a further check (*SI Appendix*, Fig. S3), we computed how often the correlation coefficient in the coarse-resolution model is smaller than that in the SRM. For the vast majority of rainy points (61.5%), this happens at best 10% of the time, meaning 6 y out of 60 y, confirming that the feedback is consistently weaker in the storm-resolving simulation. The areas where the coarse-resolution model shows frequent smaller coefficient mostly belong to the areas where the SRM falls within one SD of the year-to-year variability of the correlation coefficient of the coarse-resolution model.

**Fig. 2.**
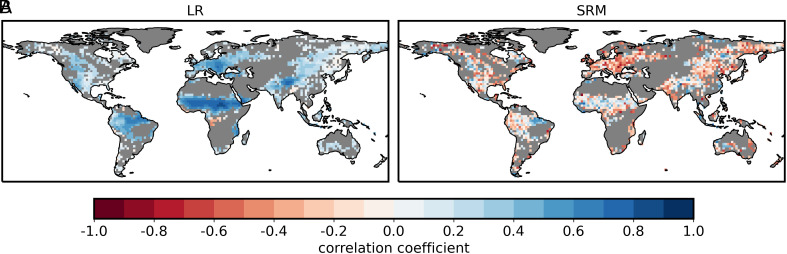
Correlation coefficient between SMI and subsequent 9-d mean precipitation for (*A*) the 60-y mean of the coarse-resolution model (LR) and (*B*) the SRM. Gray areas as in Fig. 1.

It is not only that the strength is weaker in the SRM, but the sign also differs. In the coarse-resolution model, we observe widespread positive correlations, covering 97.9% of the points. In the SRM, those areas have shrunk to 65.8%. Part of this positive correlation could result from the fact that days with precipitation tend to have higher soil moisture simply because precipitation replenishes soil moisture. To filter out this effect, we follow Duerinck et al. (43) and compute the correlation coefficient between SMI and subsequent 9-d mean precipitation (Fig. 2). In the SRM, this changes the sign of the correlation coefficient to widespread negative (66.6% of the points), whereas the values in the coarse-resolution model remain mostly unaffected. Using different rain averaging periods (*SI Appendix*, Fig. S4) does not alter this behavior.

Although our study focuses on boreal summer, due to the larger frequency of convective events over land, repeating the analysis for austral summer (*SI Appendix*, Fig. S5) reveals broadly consistent results. The feedback is weaker in the SRM. Compared to boreal summer, the differences are reduced in the northern hemisphere and enhanced in the southern hemisphere. This is consistent with the shift of frontal activity to the northern hemisphere and of convective activity to the southern hemisphere during austral summer.

### Weaker Soil Moisture–Evapotranspiration Feedback.

To understand why the soil moisture–precipitation feedback is weaker and even negative in the SRM, we decompose the feedback into its terrestrial and atmospheric segments by computing correlation coefficients between SMI and evapotranspiration as well as between evapotranspiration and precipitation.

The strength of the soil moisture–evapotranspiration feedback (Fig. 1*D*–*F*) is weaker in the SRM than in the coarse-resolution model at 70.4% of the points. Like with the soil moisture–precipitation feedback, but to a lesser extent, the sign has also changed. In addition, 86.6% of the points exhibit positive correlations in the coarse-resolution model, against 71.3% in the SRM. A weaker or even negative soil moisture–evapotranspiration feedback means that evapotranspiration is less often soil moisture limited and rather constrained by the amount of available energy. This is confirmed by determining to which soil moisture–evaporation regime grid points belong, following the method of Budyko (44, 45) (*SI Appendix*, Fig. S6). In the coarse-resolution model, the percentages are 6.5% for dry regime, 81.6% for transitional regime, and 11.9% for wet regime. The corresponding numbers are 3.5%, 59.5%, and 37% for the SRM.

**Fig. 3.**
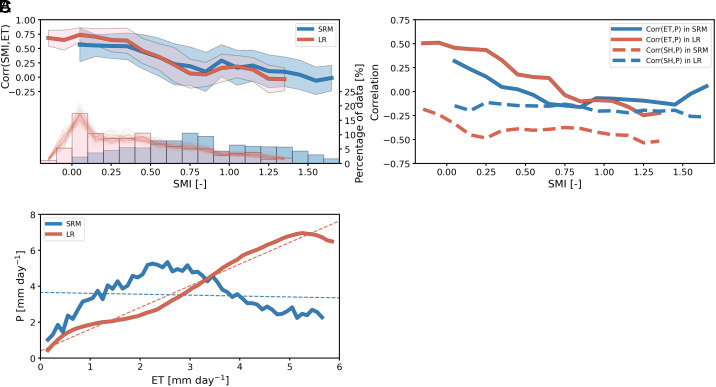
(*A*) Correlation coefficient based on daily mean values for June–July–August between SMI and evapotranspiration (ET) as a function of SMI for the 60-y mean of the coarse-resolution (red line) and the storm-resolving (blue line) models with ±1 SD in shading together with histogram of 60-y mean (bar) and each year (thin red line) of SMI (%). Bin with less than 1% of data are excluded. (*B*) Same as (*A*) but correlation coefficient between evapotranspiration and precipitation (P) (solid line) and between sensible heat flux (SH) and precipitation (dashed line). (*C*) Precipitation as a function of evapotranspiration (solid line) with linear regression (dashed line) for the two models. Data are excluded if the percentage of data per bin is less than 0.3% of total.

The difference in feedback between the two models may stem from a difference in the functional relationship between soil moisture and evapotranspiration or from different soil moisture amounts. Fig. 3*A* indicates that the strength of the correlation between SMI and evapotranspiration exhibits very similar values across soil moisture indices in both models. This similarity is consistent with the fact that the two models employ the same land surface model, the same soil and vegetation properties except for the leaf area index, see methods and *SI Appendix*, Fig. S7. The existing differences in leaf area index between the two models nevertheless do not match well with the obtained differences in the soil moisture–evapotranspiration correlation coefficient between the two models. For instance, differences in the correlation coefficient over Europe or over Africa in the northern hemisphere are always of the same sign, whereas differences in leaf area index exhibit both positive and negative values within each domain. Hence, we attribute the weaker soil moisture–evapotranspiration feedback in the SRM primarily to the occurrence of higher soil moisture, as confirmed by Fig. 3*A*. The two soil moisture distributions are distinct, with the distribution from the SRM being shifted to higher values, independently of the years included in the coarse-resolution model (see thin lines in Fig. 3*A*). In the SRM, soil moisture indices are higher than 0.5 at 75.6% of the points, whereas this number decreases to only 37.6% for the 60-y mean in the coarse-resolution model. Likewise, if we take 75% of the field capacity as the critical soil moisture value for the start of the wet regime, we find that soil moisture is larger than this value 37% of the times in the SRM, versus 11.9% in the coarse-resolution model. Higher soil moisture values are already present at the beginning of the boreal summer season (*SI Appendix*, Fig. S8 *A*–*C*), and then, soil moisture remains higher in the SRM during the analysis period (*SI Appendix*, Fig. S1) and most regions even moistened during the summer season, ending up at a higher soil moisture value (*SI Appendix*, Fig. S8 *D*–*F*). This may indicate the tendency of the SRM to equilibrate at higher soil moisture than the coarse-resolution model. That the two models would equilibrate at a different soil moisture would be consistent with the two models having a distinct land–atmosphere coupling and with the fact that simulations with and without convective parameterization rain differently (see figure 9 in Prein et al. 46).

### Negative Evapotranspiration–Precipitation Feedback.

Looking now at the atmospheric segment, the coarse-resolution model exhibits expected positive correlations between evapotranspiration and precipitation. In contrast, in the SRM, those correlations are changed to negative ones (Fig. 1*G*–*I*). In the coarse-resolution model, the sign of the feedback is positive for 72.6% of the points. Negative signs can only be found over the regions in the wet regime (compare to *SI Appendix*, Fig. S6*A*), those being North-Eastern America and Eastern Asia. In the wet regime, evapotranspiration is primarily determined by net radiation and tends to decrease when precipitation increases due to increased cloud cover (*SI Appendix*, Fig. S9). In contrast, in the SRM, negative feedback exists at 56.2% of the points. Even over the region with remaining positive feedback, the strength is weaker, with a value of 0.22 compared to 0.35 in the coarse-resolution model on average.

Two mechanisms could explain the weaker and more negative correlations in the SRM: more wet regimes and/or a distinct relationship between evapotranspiration and precipitation. As already indicated in Section 2, wet regimes are much more widespread in the SRM than in the coarse-resolution model. But also the functional relationship between evapotranspiration and precipitation differs: for a given SMI, the correlation between evapotranspiration and precipitation is weaker in the SRM where SMI is smaller than 0.9 (Fig. 3*B*). More strikingly (Fig. 3*C*), whereas precipitation linearly increases with evapotranspiration in the coarse-resolution model, there is no monotonic increase in the SRM. This suggests that evapotranspiration is not the main control of precipitation in the SRM, also evidenced by the fact that it can still rain by very small evapotranspiration amounts, in contrast to the coarse-resolution model.

Hohenegger et al. (27) explained the distinct feedback sign between parameterized and explicit convection in their limited-area simulations over the Alpine region by the design of the triggering function of their default convective parameterization, making convection couples to moisture rather than to heat. A similar mechanism could be at play here. At least, the correlation between sensible heat flux and precipitation is indeed stronger in the SRM than in the coarse-resolution model for a given SMI (Fig. 3*B*). Moreover, in the presence of soil moisture heterogeneities, Taylor et al. (30) showed that afternoon precipitation is triggered over dry soils, while coarse-resolution models favor wet soils. In our SRM, afternoon precipitation is indeed triggered over dry soils (*SI Appendix*, Fig. S10), as in observations (see figure 1 in Taylor et al. 14 and figure 1A in Guillod et al. 47). These results could further explain the documented weaker evapotranspiration–precipitation feedback in the SRM. Finally, it may just be an indication that the SRM couples convection to circulation (48) rather than to surface fluxes.

### Land–Atmosphere Coupling in Observations.

In the previous sections, we demonstrated that the coupling between the land surface and the atmosphere is weaker and more negative in the SRM. Do observations agree with our findings? To determine which model better represents the coupling, we compare the simulated coupling to the one recorded by the FLUXNET2015 dataset (49) at 102 sites (*Materials and Methods*). Although being observations, it is important to remember that those observations still contain measurement errors and noise that will degrade the correlation (50). The comparison also suffers from the different scales considered: point observation is ideally representative for a larger fetch area versus a km-scale and a low-resolution model output downgraded to a 2^°^ grid analysis box.

**Fig. 4.**
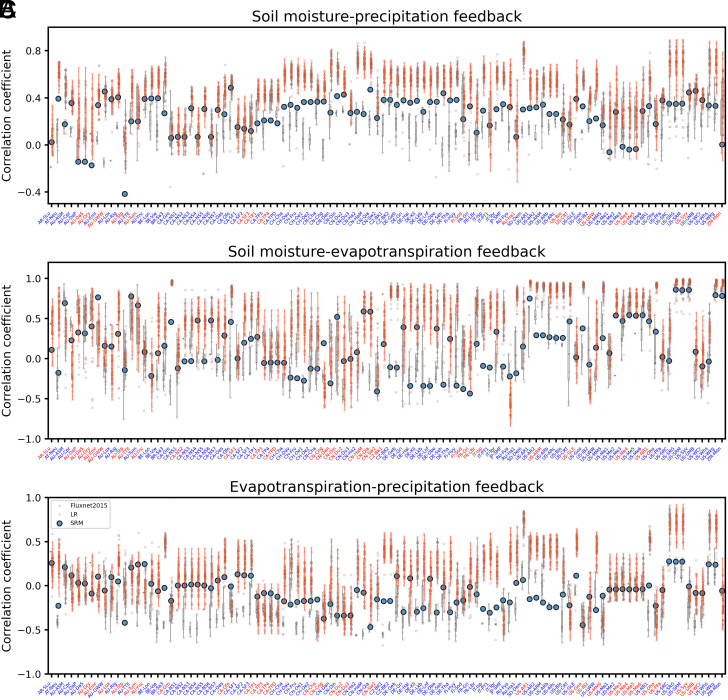
Correlation coefficients computed based on daily mean values for June–July–August between (*A*) soil moisture and precipitation, (*B*) soil moisture and evapotranspiration, and (*C*) evapotranspiration and precipitation for the 60-y data of the coarse-resolution model (LR, red), the SRM (blue), and FLUXNET2015 (gray) at each site. The site names are colored in blue when SRM is closer to FLUXNET2015 and colored in red when LR is closer to FLUXNET2015. Center line: median; box limits: 25th and 75th percentiles; whiskers: ±1.5 times the interquartile range from the box limits; dots: correlation coefficient for each year. Which measurement depths or model soil layer depths are used to compute correlation coefficients at each site are listed in *SI Appendix*, Table S1.

Fig. 4 provides clear evidence that the SRM better represents the sign and strength of land–atmosphere coupling. In terms of the soil moisture–precipitation feedback, the SRM exhibits closer values to FLUXNET2015 at 83.3% of sites. Strikingly, the correlation coefficients derived from the coarse-resolution model rarely fall into the range of the correlation coefficients measured at the selected FLUXNET2015 sites. Similarly, for the soil moisture–evapotranspiration and evapotranspiration–precipitation feedback, the SRM indicates closer correlations to FLUXNET2015 at 71.6% and 70.7% of the sites, respectively.

## Concluding Thoughts

Our study shows that land–atmosphere coupling in a global SRM is weaker than what we have known from global coarse-resolution models and is much closer to observations. Not only the feedback between soil moisture and evapotranspiration is weaker, but the feedback between evapotranspiration and precipitation as well as between soil moisture and precipitation is weaker. The preference is for a negative soil moisture–precipitation feedback, against a positive feedback in global coarse-resolution models. The underlying hypothesis is that this difference primarily stems from the treatment of convection, being parameterized at the coarse resolution but explicit at the storm-resolving resolution, rather than by other differences in the employed model setups such as simulated period, grid spacing, and treatment of the land surface. To test this hypothesis, we recomputed the correlation coefficient between soil moisture and precipitation and their difference to the SRM in a coarse-resolution model under present-day conditions instead of pre-industrial, with two different grid spacings (180 vs. 100 km), using the wettest year instead of the mean, and using a different treatment of plant (*SI Appendix*, Fig. S11). Despite some variation in the strength of the feedback, all coarse-resolution simulations exhibit positive feedback and feedback that is systematically stronger than at storm-resolving resolution. This is consistent with past literature which has revealed a positive feedback at coarse resolution despite the use of various climate models. Finally, we repeated our analysis on a newer version of our 5-km model. This newer version especially entails an updated description of land-surface fields, which makes it less comparable to our coarse-resolution model, but this version was integrated globally, coupled to an ocean, for five full years (*SI Appendix*, Fig. S12). Every year exhibits predominantly weaker correlation coefficients between soil moisture and precipitation in the storm-resolving than in the coarse-resolution model, confirming our results.

Our results imply the possibility that global coarse-resolution models, due to too strong and too positive coupling, may overestimate the increase in frequency/strength of droughts and heatwaves under climate change. Moreover, the results of this study may suggest that precipitation over the Amazon is more resilient to deforestation than previously thought. This is so because a decrease in evapotranspiration following deforestation should not lead to a strong decrease in precipitation in a model with weak and negative feedback between soil moisture, or evapotranspiration, and precipitation. Also, as weak feedback between soil moisture and precipitation implicitly means that soil moisture is less important for the atmosphere, the state of the soil moisture, being wet or dry, especially at the initial time, and related spin-up issues, may be of less relevance at storm-resolving than at coarse resolution. Our study highlights that global SRMs can behave fundamentally different than state-of-the-art coarse-resolution models, demonstrating the needs to consider such models for studies of climate change over land.

## Materials and Methods

### Simulations.

We use two simulations integrated with different configurations of the ICON model. The first one is a fully coupled global simulation integrated at 5 km. This simulation was initialized on 20 January 2020 and integrated until 28 February 2021. It employs the new configuration of the ICON model, called Sapphire and is fully described in Hohenegger et al. (40), see the simulation called G_AO_5km. The second simulation is a fully coupled global simulation integrated at 160 km (42). It is part of the CMIP6 and we use the piControl simulation as it allows a better estimation of interannual variability than a transient simulation such as the historical simulation. The official simulation name is ICON-ESM-LR.piControl (51). We limit our analysis to a 60-y of the simulation as extending the time span of the data does not change the main findings.

The key difference between the two simulations is that convection is explicitly resolved at 5 km but parameterized at 160 km in ICON-ESM-LR.piControl. Both simulations use JSBACH (52) to represent land surface processes, with five soil layers, but slightly different configurations for the vegetation processes. Given the shortness of the simulation, the big leaf (BL) approach is used at 5 km, whereas plant functional types (PFT) approach is used in ICON-ESM-LR.piControl. The main differences between the two approaches are i) BL does not explicitly define a vegetation type, PFT up to eleven types of vegetation per grid box, and ii) BL prescribes leaf area index from monthly-mean observed climatological values, whereas PFT computes leaf area index interactively based on phenology. The other soil and vegetation properties are based on the same datasets.

### Coupling Metric.

To quantify the strength and the sign of land–atmosphere coupling, first, the simulation output is daily averaged and, second, interpolated to a common 2^°^ grid. Using a larger box of, for instance, 5^°^ grid or 10^°^ grid, does not affect the main conclusion of the study. Lastly, we compute three Pearson correlation coefficients between: i) SMI and precipitation, ii) SMI, and evapotranspiration, and iii) evapotranspiration and precipitation. The analysis is restricted to the boreal summer season (June–July–August, JJA). For the soil moisture analysis, we use the SMI as this is the variable employed in the computation of evapotranspiration in JSBACH:

[1] $$\begin{array}{c} \;\text{SMI}=\frac{\theta -\theta_{\;\text{pwp}}}{\theta_{\;\text{crit}}-\theta_{\;\text{pwp}}}, \end{array}$$

where θ is soil moisture in the root zone, $\theta_{\;\text{pwp}}$ is the permanent wilting point, and $\theta_{\;\text{crit}}$ is 75% of the field capacity. These definitions follow what is done in JSBACH.

Given the 60-y time span of the coarse-resolution model, we compute the correlation coefficients for each year and then average them over 60 y. Subsequently, we only retain the computed correlations if the JJA mean precipitation is larger than 0.1 mm d^−1^ in both simulations and where differences in the correlation coefficient between SMI and precipitation between the two models may not be due to internal variability. We assess this by computing the SD of the correlation coefficient between SMI and precipitation in the coarse-resolution model based on the 60 y of simulation output and only retain the points where the difference in correlation coefficient between the two models is larger than one SD.

As a further metric, we compute the correlation coefficient between SMI and subsequent precipitation. It is computed following Duerinck et al. (43) by correlating daily mean SMI with precipitation averaged on subsequent days. We tested the sensitivity to the chosen averaging period by varying it between 1 and 120 d, see *SI Appendix*, Fig. S4.

### FLUXNET2015.

We use the FLUXNET2015 dataset (49) to evaluate the simulated coupling. Among 206 available sites, we select 102 sites which have measurements of daily soil moisture, precipitation, and latent heat flux, needed to compute correlation coefficients. Days when at least one of the variables exhibits a quality control flag with a value below 0.75 are filtered out, conservatively. Since soil moisture in the root zone is not available at all sites, we compute correlation coefficients using surface soil moisture in FLUXNET2015 and simulated soil moisture in the soil layer closest to the measurement depth at each site for the simulations. Which measurement depths or soil layer depths are used to compute the correlation coefficients at each site are listed in *SI Appendix*, Table S1.

## Supplementary Material

Appendix 01 (PDF)

## Data Availability

The G_AO_5km simulation was done with the ICON branch nextgems_cycle1_dpp0066 as commit62dbfc (https://doi.org/10.17617/3.1XTSR6 (40)). The ICON-ESM-LR.piControl simulation is available here https://www.wdc-climate.de/ui/cmip6?input=CMIP6.CMIP.MPI-M.ICON-ESM-LR.1pctCO2 (51). FLUXNET2015 data can be accessed at https://fluxnet.org/data/fluxnet2015-dataset/ (49). The key analysis codes can be found from GitHub (https://github.com/junhonglee89/PNAS_land-atm_coupling_in_global_SRM) (53).
