# Supplementary material for: Weaker land–atmosphere coupling in global storm-resolving simulation
Source: Proc Natl Acad Sci U S A. 2024 Mar 12;121(12):e2314265121. doi: 10.1073/pnas.2314265121 (PMC10962945; doi:10.1073/pnas.2314265121)
Supplement: Supplementary file 1 — Appendix 01 (PDF) [file pnas.2314265121.sapp.pdf]

Supporting Information for

Weaker land-atmosphere coupling in global storm-resolving simulation

Junhong Lee, Cathy Hohenegger

Corresponding Author: Junhong Lee
E-mail: junhong.lee@mpimet.mpg.de

This PDF file includes:

Supporting text
Figs. S1 to S12
Table S1
SI References

Supporting Information Text

A. Soil moisture-evaporation regime

Previous studies (1, 2) have classified the dependency between evapotranspiration and soil moisture into three regimes: dry, transitional, and wet. Following these studies, we define those as: dry regime ($\theta <= \theta_{\mathrm{pwp}}$), transitional regime ($\theta_{\mathrm{pwp}} < \theta < \theta_{\mathrm{crit}}$), and wet regime ($\theta >= \theta_{\mathrm{crit}}$), where θ is volumetric soil moisture in the root zone, θ_{pwp} is the permanent wilting point, and θ_{crit} is critical soil moisture (see method). For this analysis, we average soil moisture over June-July-August first and then determine the regime. In the dry regime, soil moisture is smaller than the permanent wilting point, and thus evapotranspiration should be constant and close to zero. In the intermediate regime, soil moisture limits evapotranspiration and evapotranspiration should increase with soil moisture, independently of the amount of available energy at the surface. In the wet regime, there is enough soil moisture and evapotranspiration is limited by the availability of net energy.

B. Preference for afternoon precipitation over soil moisture anomalies

Taylor et al. (3) and follow-up studies (4, 5) have analyzed whether rain is more likely to occur over soils wetter or drier than their surrounding. We apply their approach to the output of our storm-resolving simulation. First, precipitation and soil moisture index are interpolated onto a common grid of 0.25°. Then, an 1.25° by 1.25° event domain (L_{evt}) is defined, centered at a cell location (L_{max}) which coincides with a maximum in afternoon rainfall. Afternoon rainfall is defined as rainfall accumulated from 12:00 to 24:00. One or more cells are defined as L_{min} where afternoon rainfall is at a minimum within L_{evt}. In this step, we exclude L_{evt} if it contains a cell with 1) morning precipitation (06:00-12:00) exceeding 1 mm day^{-1}, 2) topographic height variability exceeding 300 m, and 3) water bodies. We then compute morning (06:00-12:00) soil moisture index anomalies (S) by subtracting 31-day moving averaged soil moisture index centered at corresponding day. We compute the difference in soil moisture anomaly between L_{max} and L_{min} for event days as event sample, which is denoted as ΔS_{e}. As the next step, we compute the difference in S between L_{max} and L_{min} using data from the same calendar month but for non-event days, denoted ΔS_{c}. This builds our control sample.

Afterward, all event and control samples within each 5° by 5° box are pooled together. The statistical significance is evaluated by assessing whether the differences between the two samples are significantly larger or smaller than the randomly generated differences. Before evaluating the significance, the climatology of the individual 5° boxes is removed by subtracting June-July-August mean. Then, we compute $\delta_{\mathrm{e}} = \mathrm{mean}(\Delta S_{\mathrm{e}}) - \mathrm{mean}(\Delta S_{\mathrm{c}})$ as the difference between event and control samples. For the randomly generated difference, we compute $\delta = \mathrm{mean}(\Delta \hat{S}_{\mathrm{e}}) - \mathrm{mean}(\Delta \hat{S}_{\mathrm{c}})$ where $\Delta \hat{S}_{\mathrm{e}}$ is randomly pooled sample (the same size of the event sample) from ΔS_{e} and ΔS_{c}, and $\Delta \hat{S}_{\mathrm{c}}$ is randomly pooled sample from the remainder, and we repeat the computations 1,000 times (bootstrapping). Finally, we quantify the percentile of δ_{e} with respect to δ. For a more detailed description of the approach, please refer to previous studies (3–5).

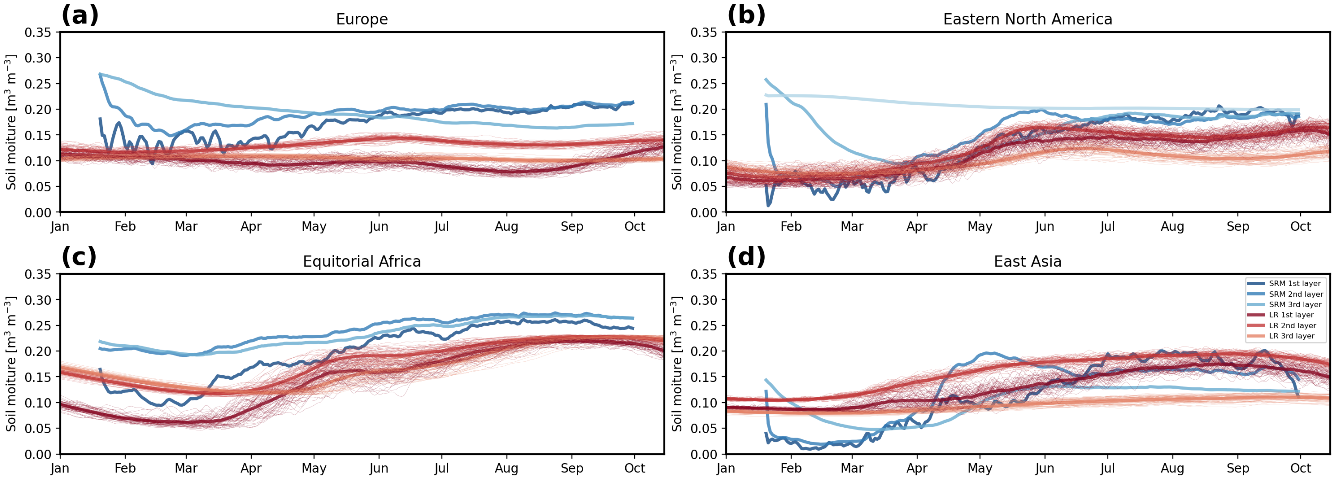

Fig. S1. Time series of soil moisture at each soil layer averaged over (a) Europe (36°N-72°N and 11°W-46°E), (b) Eastern North America (35°N-55°N and 98°W-60°W), (c) Equatorial Africa (2°S-13°N and 18°W-40°E), and (d) East Asia (35°N-55°N and 90°E-145°E) for 60-year mean (thick red) and individual year (thin red) of the coarse-resolution model (LR), and the storm-resolving model (SRM, thick blue).

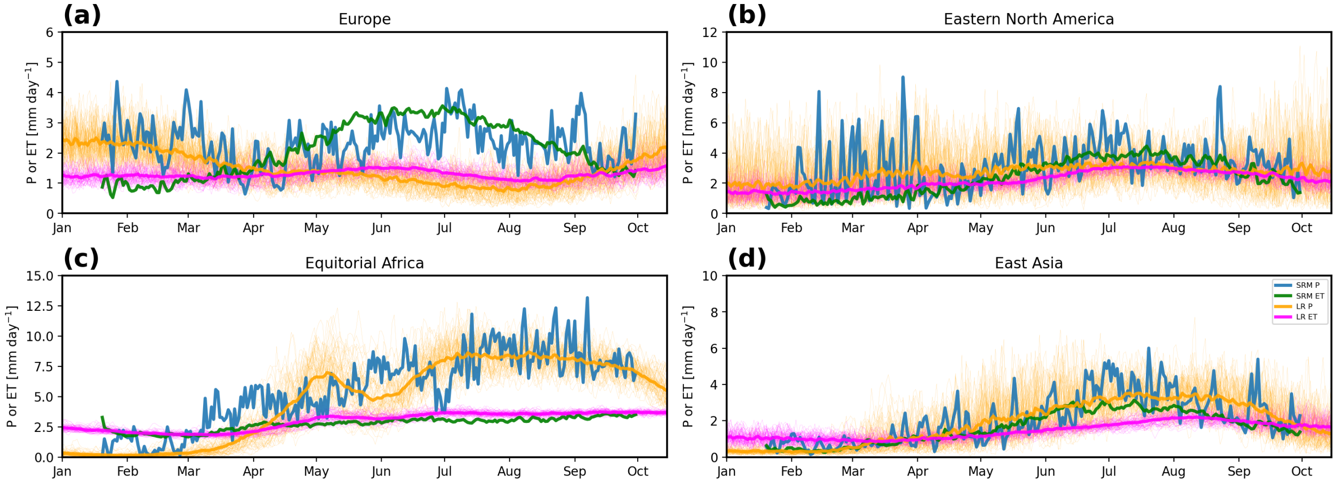

Fig. S2. Same as Fig. S1 but for time series of precipitation (P) and evapotranspiration (ET) for 60-year mean (thick orange for P and magenta for ET) and individual year (thin orange for P and magenta for ET) of the coarse-resolution model (LR), and the storm-resolving model (SRM, blue for P and green for ET).

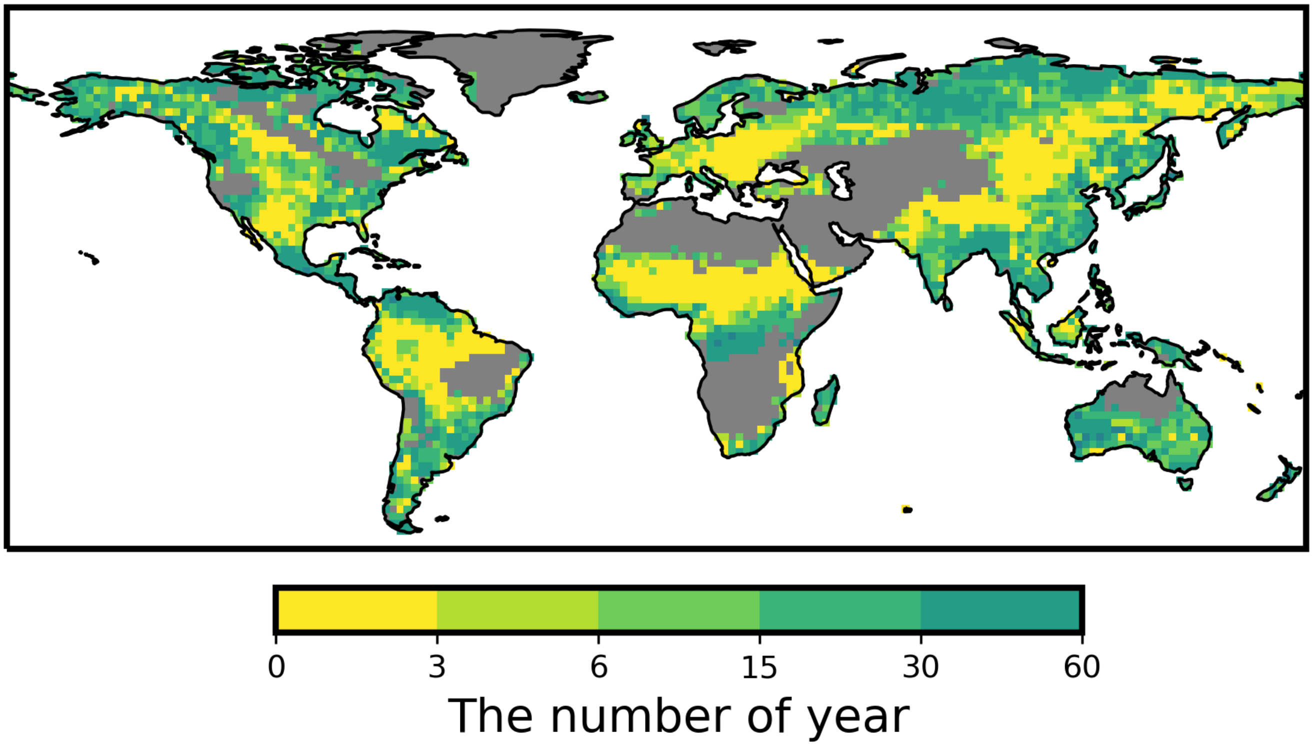

Fig. S3. The number of year when the correlation coefficient between soil moisture index-precipitation in the coarse-resolution model is smaller than that in the storm-resolving model. Areas where precipitation is smaller than 0.1 mm day^{-1} in both simulations are masked in grey.

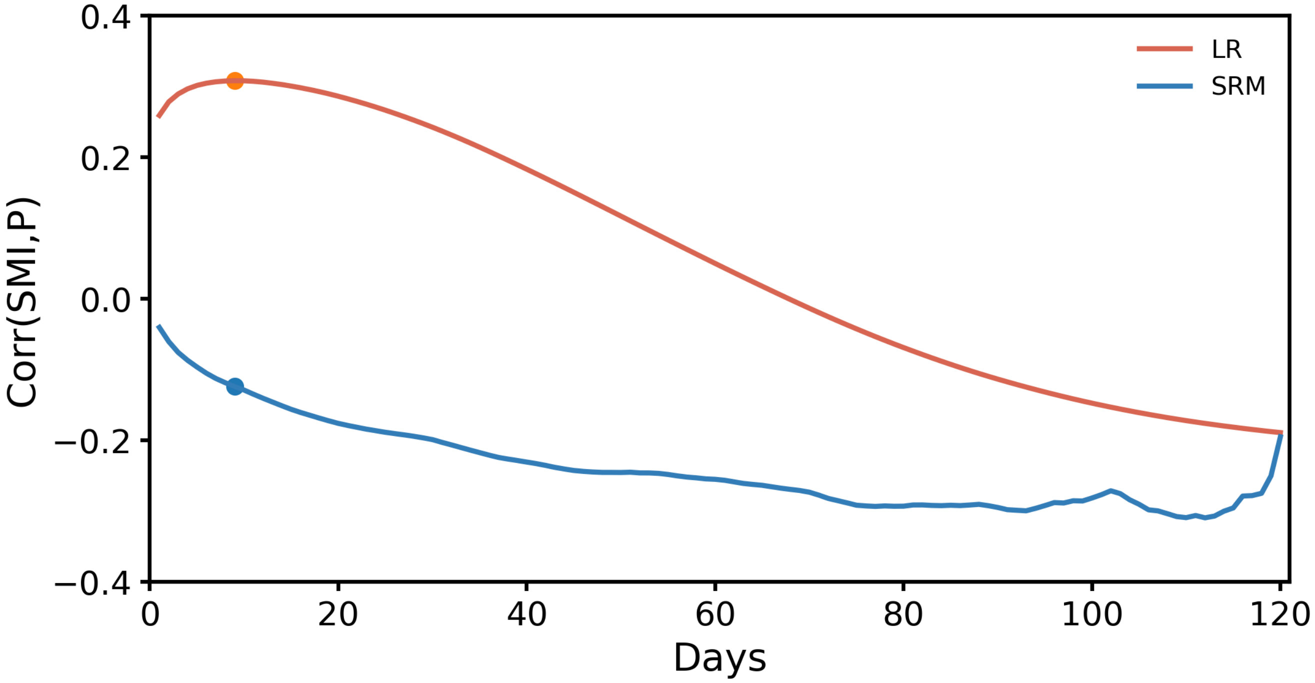

Fig. S4. Dependency of the correlation coefficient between soil moisture index (SMI) and subsequent mean precipitation (P) on the averaging period chosen to compute the mean precipitation for the coarse-resolution model (LR, red) and the storm-resolving model (SRM, blue). Dots indicate the averaging period of 9 days when the correlation coefficients for LR reach the maximum.

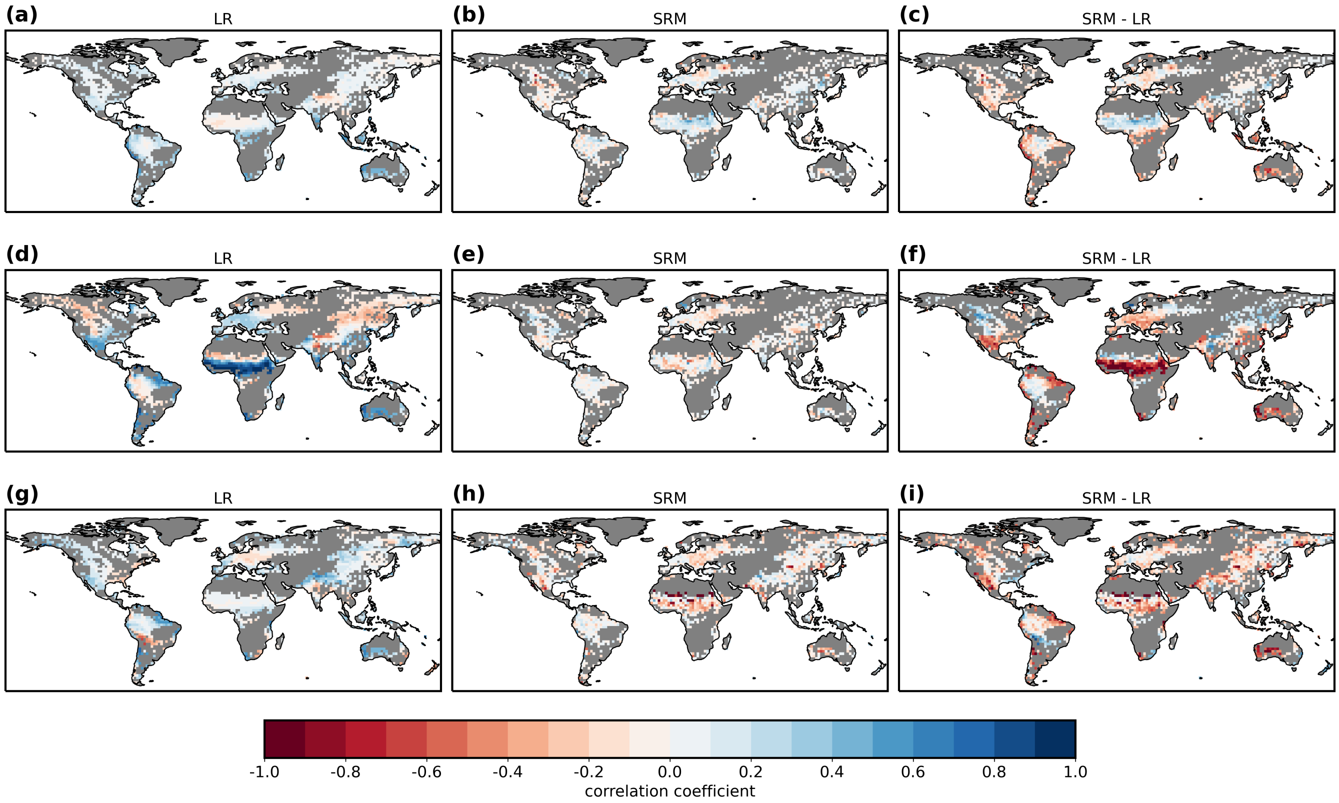

Fig. S5. Correlation coefficients computed based on daily mean values for December-January-February between (a-c) soil moisture index and precipitation, (d-f) soil moisture index and evapotranspiration, and (g-i) evapotranspiration and precipitation for the 60-year mean of the coarse-resolution model (LR), the storm-resolving model (SRM), and the resulting difference in correlation coefficient. Areas where precipitation is smaller than 0.1 mm day^{-1} in both simulations or where the soil moisture index-precipitation correlation coefficient of the storm-resolving model is within one standard deviation of the year-to-year variability of the correlation coefficient in the coarse-resolution model are masked in grey.

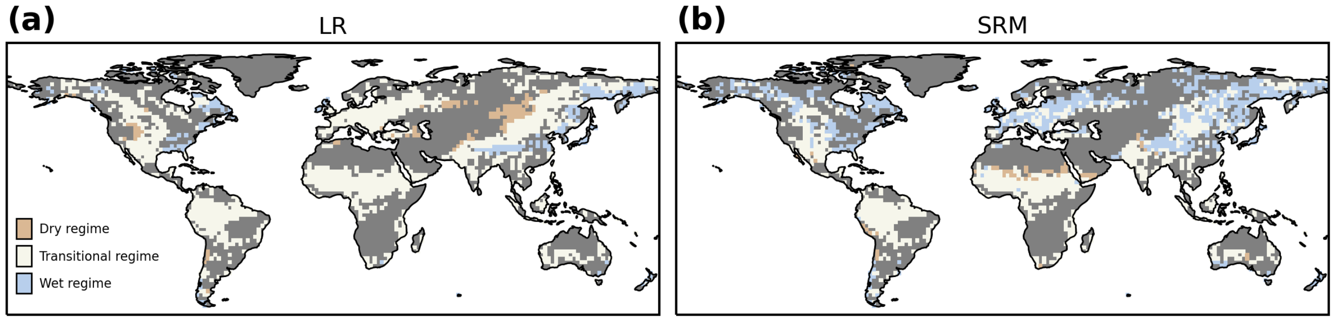

Fig. S6. Soil moisture-evaporation regime for (a) the coarse-resolution model (LR) and (b) the storm-resolving model (SRM). See definition in section A of SI Appendix.

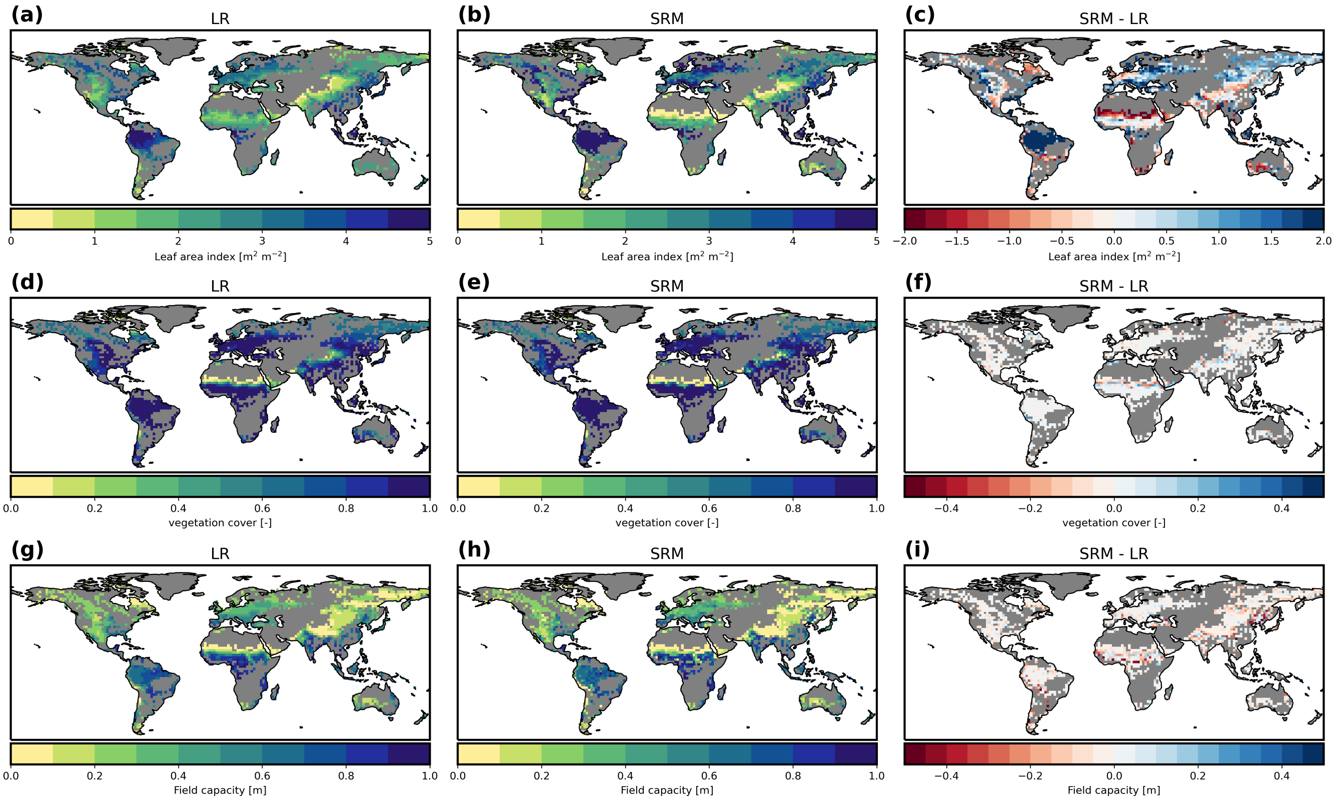

Fig. S7. (a-c) Leaf area index, (d-f) vegetation cover, and (g-i) field capacity for the coarse-resolution model (LR), storm-resolving model (SRM), and their difference. The averaging period for leaf area index is 60 years for LR and one year for SRM during June-July-August. With the exception of the leaf area index, two key soil and vegetation properties used for the computation of evapotranspiration in ICON are similar between the two models.

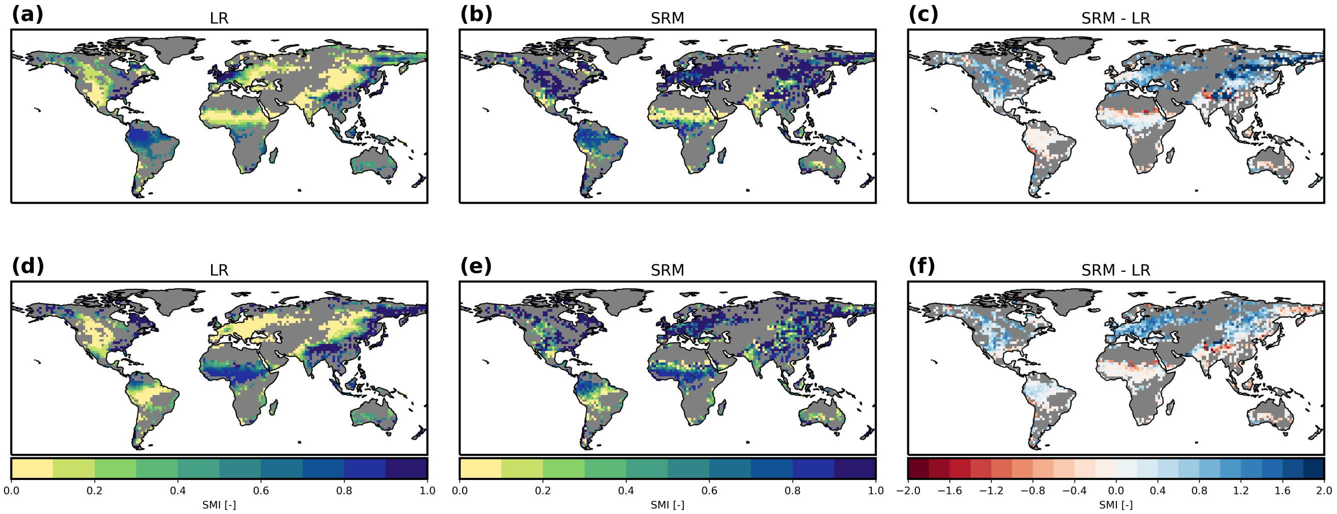

Fig. S8. Soil moisture index (a-c) on 1 June and (d-f) on 1 September for (left) 60-year mean of the coarse-resolution model (LR), (middle) the storm-resolving model (SRM), and (right) their difference.

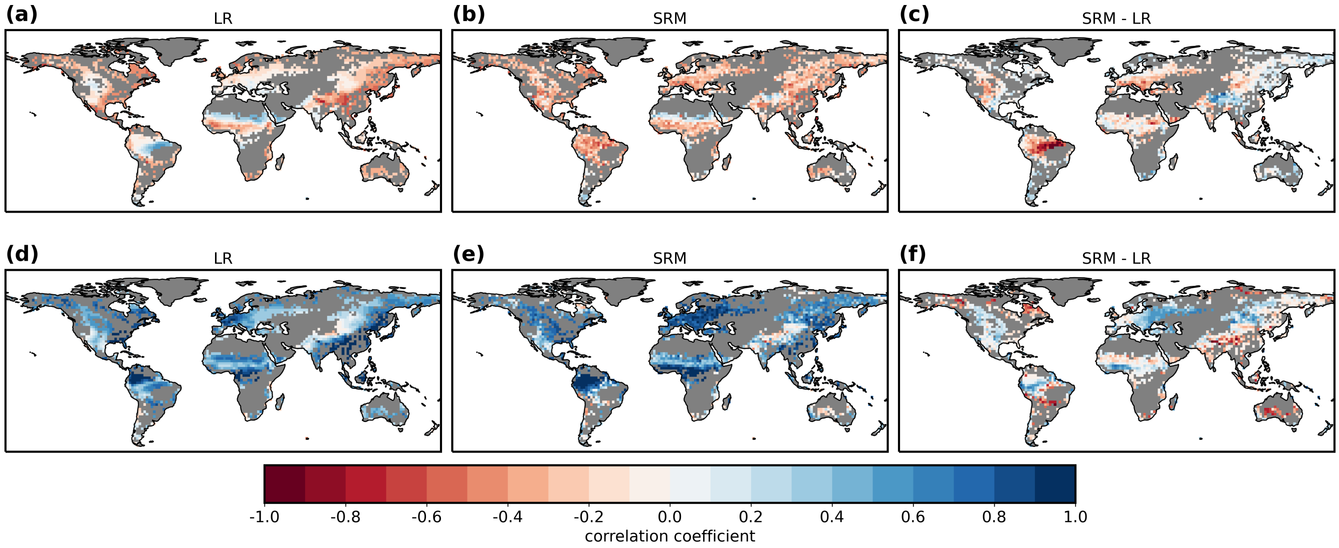

Fig. S9. Correlation coefficient computed based on daily mean values for June-July-August between (a-c) precipitation and surface net radiation and (d-f) evapotranspiration and surface net radiation for (left) the 60-year mean of the coarse-resolution model (LR), (middle) the storm-resolving model (SRM), and (right) their difference. Gray areas as in Fig. S5.

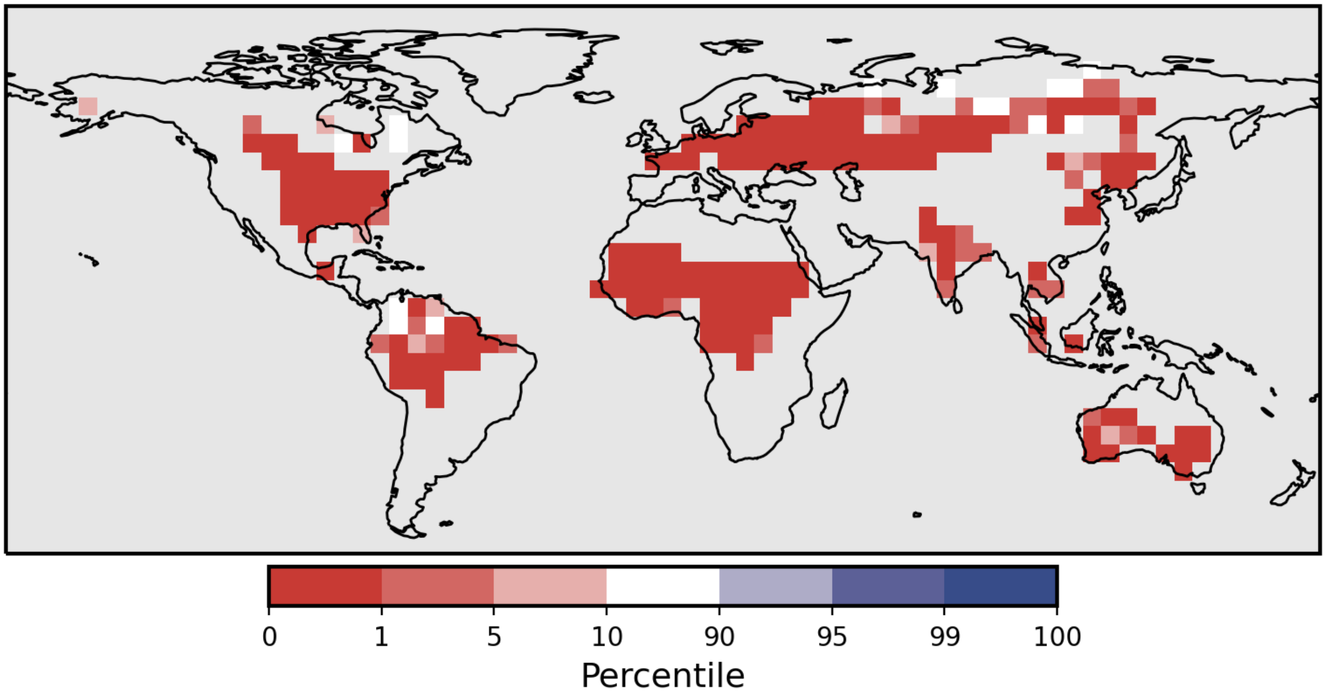

Fig. S10. Percentiles of δ_e with respect to δ indicating the preference for afternoon precipitation over soil moisture anomalies for June-July-August in the storm-resolving model (see definition in section B of SI Appendix). The lower percentile denotes that afternoon precipitation is triggered over dry soils and the higher percentile means afternoon precipitation is triggered over wet soils. Gray area denotes 5° x 5° cells containing fewer than 25 events sample.

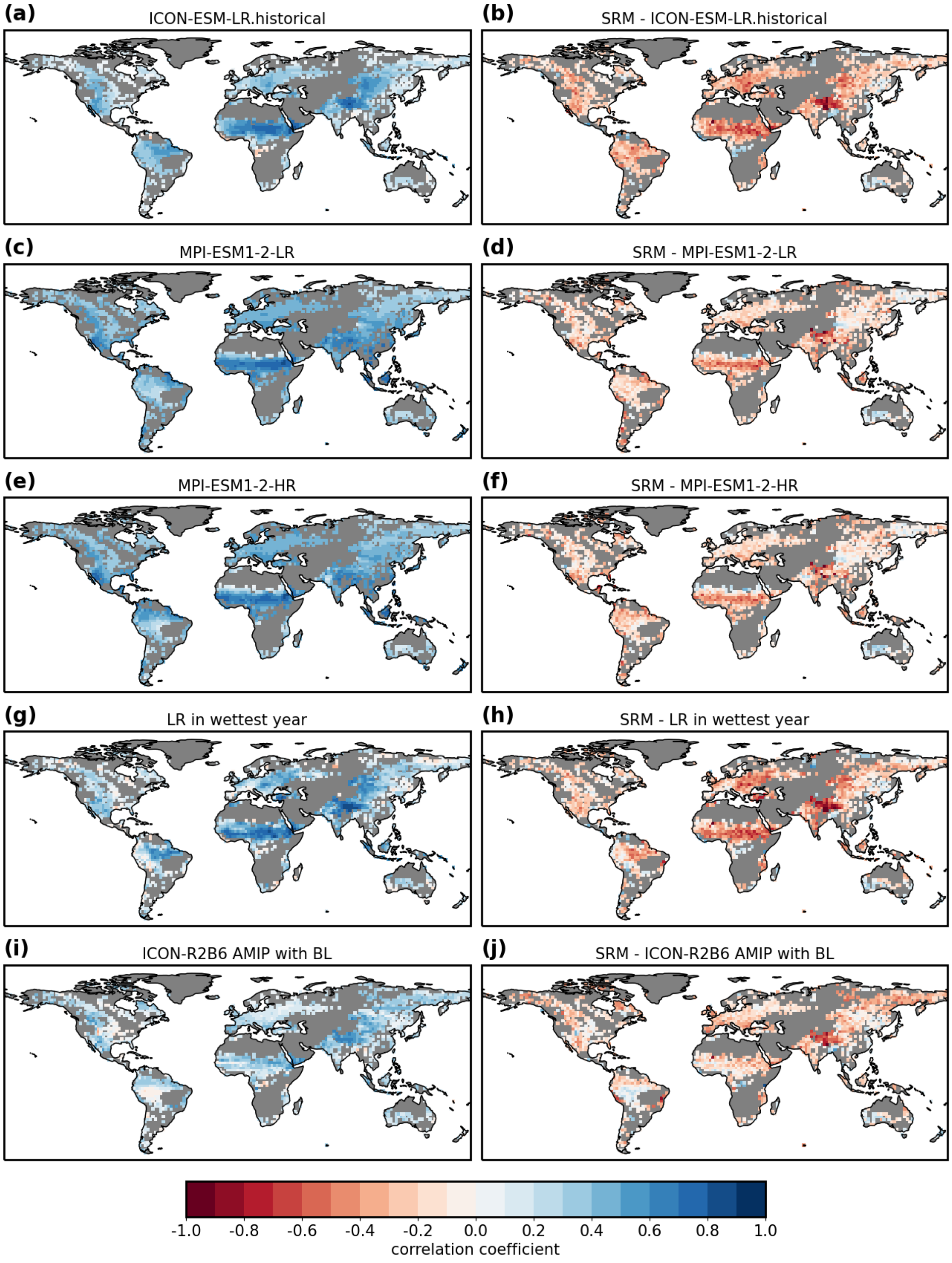

Fig. S11. Correlation coefficient between soil moisture and precipitation computed based on daily mean values for June-July-August and its difference from the storm-resolving model (SRM) for (a, b) ICON-ESM-LR.historical, which uses the same model version with the coarse-resolution model (LR, ICON-ESM-LR.piControl) but we use historical and the latest 15 years, to have years closer to present day, which is the year simulated in SRM and thus test whether the climate matters; (c-f) MPI-ESM1-2-LR at T64 (about 180 km) and MPI-ESM1-2-HR at T127 (about 100 km) for 30 years which use MPI-ESM that it is the predecessor of ICON-ESM and it shares the same land surface scheme and physical scheme, and we use them to test the effect of grid spacing but did not have two ICON-ESM runs we different resolution so that why we used MPI-ESM; (g-h) the wettest year in LR, determined by the global mean soil moisture in June-July-August, and we use this to test whether using just one year, and one year which is wet, making it more similar to SRM, could explain the differences; and (i-j) a simulation (ICON-R2B6 AMIP) with the big leaf (BL) approach to test the effect of BL approach versus plant functional types (PFT) approach, but the simulation is Atmospheric Model Intercomparison Project (AMIP) type simulation with prescribed SST and higher grid spacing, which makes it more difficult to interpret. Top soil moisture is used to compute the correlation coefficient for two MPI-ESM simulations, as the field capacity and the permanent wilting point are not provided. Otherwise, soil moisture index in the root zone is used.

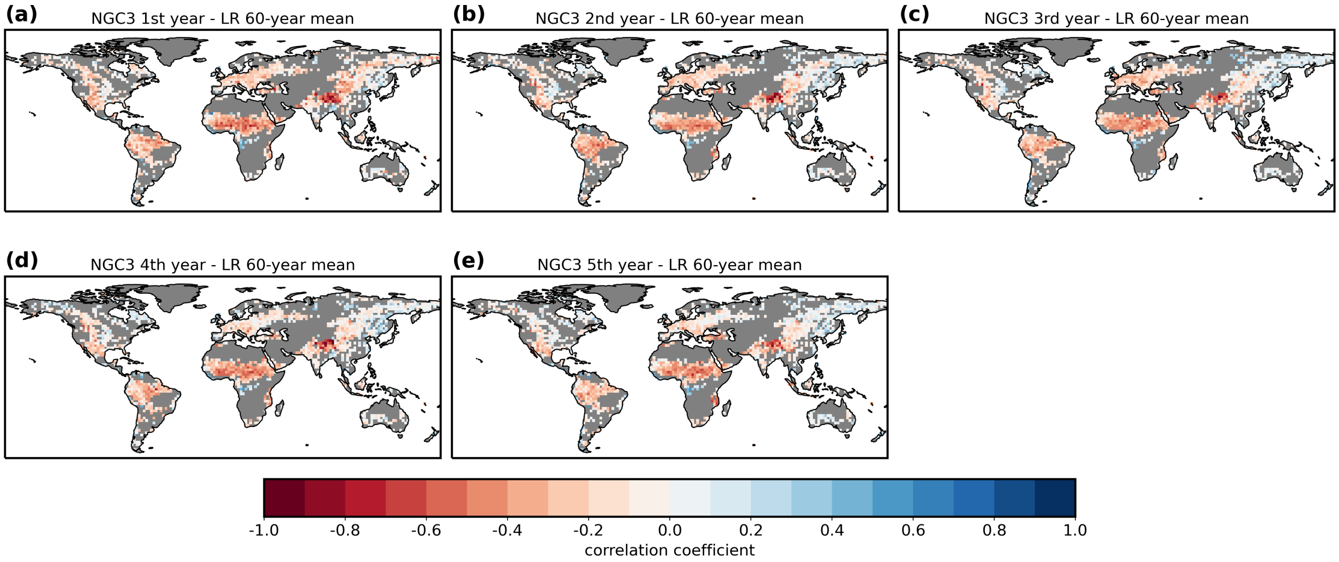

Fig. S12. Difference in the soil moisture index-precipitation coefficient between the new storm-resolving simulation (NGC3) for 5 years with a newer version of ICON model that uses difference land surface input parameters, which makes it less comparable, and the coarse-resolution model, computed across five years of the new storm-resolving model in comparison to the 60-year mean of the coarse-resolution model.

Table S1. The descriptions of selected 102 FLUXNET2015 sites and corresponding soil layer depths of soil moisture for the coarse-resolution and storm-resolving models, used to compute correlation coefficients in Fig. 4.

Station name	Longitude(°)	Latitude(°)	Measurement depth (cm)	Soil layer depth of model (cm)
AR-SLu	-66.46	-33.465	50.0	77.55
AT-Neu	11.318	47.117	5.0	3.25
AU-ASM	133.25	-22.283	5.0	3.25
AU-Cpr	140.59	-34.002	10.0	3.25
AU-DaP	131.32	-14.063	5.0	3.25
AU-DaS	131.39	-14.159	5.0	3.25
AU-Dry	132.37	-15.259	5.0	3.25
AU-Emr	148.47	-23.859	5.0	3.25
AU-GWW	120.65	-30.191	5.0	3.25
AU-Lox	140.66	-34.47	5.0	3.25
AU-Rig	145.58	-36.65	5.0	3.25
AU-Stp	133.35	-17.151	50.0	77.55
AU-TTE	133.64	-22.287	5.0	3.25
AU-Tum	148.15	-35.657	15.0	19.2
AU-Ync	146.29	-34.989	3.0	3.25
BE-Lon	4.7462	50.552	5.0	3.25
BE-Vie	5.9981	50.305	2.0	3.25
BR-Sa3	-54.971	-3.018	15.0	19.2
CA-Gro	-82.156	48.217	50.0	77.55
CA-NS1	-98.484	55.879	32.0	19.2
CA-NS2	-98.525	55.906	18.0	19.2
CA-NS3	-98.382	55.912	5.0	3.25
CA-NS4	-98.381	55.914	25.0	19.2
CA-NS5	-98.485	55.863	9.0	3.25
CA-NS6	-98.964	55.917	14.0	19.2
CA-NS7	-99.948	56.636	11.0	3.25
CA-Oas	-106.2	53.629	7.5	3.25
CA-Qfo	-74.342	49.693	5.0	3.25
CA-SF1	-105.82	54.485	30.0	19.2
CA-SF2	-105.88	54.254	15.0	19.2
CA-SF3	-106.01	54.092	15.0	19.2
CA-TP1	-80.56	42.661	5.0	3.25
CA-TP3	-80.348	42.707	5.0	3.25
CA-TP4	-80.357	42.71	5.0	3.25
CA-TPD	-80.558	42.635	2.0	3.25
CH-Cha	8.4104	47.21	5.0	3.25
CH-Dav	9.8559	46.815	5.0	3.25
CH-Fru	8.5378	47.116	5.0	3.25
CH-Oe1	7.7319	47.286	5.0	3.25
CH-Oe2	7.7337	47.286	5.0	3.25
CN-Cha	128.1	42.403	10.0	3.25
CN-Cng	123.51	44.593	10.0	3.25
CN-Dan	91.066	30.498	5.0	3.25
CN-Din	112.54	23.173	5.0	3.25
CN-Du2	116.28	42.047	10.0	3.25
CN-Du3	116.28	42.055	15.0	19.2
CN-Ha2	101.33	37.609	10.0	3.25
CN-HaM	101.18	37.37	5.0	3.25
CN-Qia	115.06	26.741	5.0	3.25
CN-Sw2	111.9	41.79	15.0	19.2
CZ-BK1	18.537	49.502	5.0	3.25
CZ-BK2	18.543	49.494	5.0	3.25
DE-Geb	10.915	51.1	8.0	3.25
DE-Gri	13.513	50.949	10.0	3.25
DE-Hai	10.452	51.079	8.0	3.25
DE-Kli	13.522	50.893	10.0	3.25
DE-Lkb	13.305	49.1	4.0	3.25
DE-Lnf	10.368	51.328	8.0	3.25
DE-Obe	13.721	50.787	10.0	3.25
DE-Seh	6.4497	50.871	1.0	3.25
DE-Tha	13.565	50.963	10.0	3.25

Continued on next page

Table S1. Continued.

Station name	Longitude(°)	Latitude(°)	Measurement depth (cm)	Soil layer depth of model (cm)
FI-Hyy	24.295	61.847	2.0	3.25
FI-Sod	26.639	67.362	5.0	3.25
FR-Gri	1.9519	48.844	5.0	3.25
FR-LBr	-0.7693	44.717	15.0	19.2
IT-Isp	8.6336	45.813	10.0	3.25
IT-PT1	9.061	45.201	15.0	19.2
IT-Tor	7.5781	45.844	5.0	3.25
JP-SMF	137.08	35.262	2.0	3.25
RU-Fyo	32.922	56.462	10.0	3.25
RU-Ha1	90.002	54.725	15.0	19.2
SD-Dem	30.478	13.283	5.0	3.25
US-AR1	-99.42	36.427	10.0	3.25
US-AR2	-99.597	36.636	10.0	3.25
US-ARM	-97.489	36.606	10.0	3.25
US-ARb	-98.04	35.55	10.0	3.25
US-ARc	-98.04	35.546	10.0	3.25
US-Blo	-120.63	38.895	10.0	3.25
US-CRT	-83.347	41.628	15.0	19.2
US-GLE	-106.24	41.367	5.0	3.25
US-Goo	-89.874	34.255	5.0	3.25
US-IB2	-88.241	41.841	2.5	3.25
US-LWW	-97.979	34.96	5.0	3.25
US-MMS	-86.413	39.323	15.0	19.2
US-Me1	-121.5	44.579	30.0	19.2
US-Me2	-121.56	44.452	10.0	3.25
US-Me3	-121.61	44.315	30.0	19.2
US-Me4	-121.62	44.499	30.0	19.2
US-Me5	-121.57	44.437	30.0	19.2
US-Me6	-121.61	44.323	10.0	3.25
US-NR1	-105.55	40.033	5.0	3.25
US-Oho	-83.844	41.554	15.0	19.2
US-PFa	-90.272	45.946	5.0	3.25
US-SRC	-110.84	31.908	2.5	3.25
US-SRG	-110.83	31.789	5.0	3.25
US-SRM	-110.87	31.821	5.0	3.25
US-Syv	-89.348	46.242	10.0	3.25
US-UMB	-84.714	45.56	2.0	3.25
US-WCr	-90.08	45.806	5.0	3.25
US-Whs	-110.05	31.744	5.0	3.25
US-Wkg	-109.94	31.736	5.0	3.25
ZM-Mon	23.253	-15.439	5.0	3.25

References

1. M Budyko, Heat balance of the earth's surface. gidrometeozidat (1956).
2. M Budyko, D Miller, Climate and life. academic press, new york (1974).
3. CM Taylor, RA de Jeu, F Guichard, PP Harris, WA Dorigo, Afternoon rain more likely over drier soils. *Nature* **489**, 423–426 (2012).
4. BP Guillod, B Orlowsky, DG Miralles, AJ Teuling, SI Seneviratne, Reconciling spatial and temporal soil moisture effects on afternoon rainfall. *Nat. communications* **6**, 6443 (2015).
5. H Moon, BP Guillod, L Gudmundsson, SI Seneviratne, Soil moisture effects on afternoon precipitation occurrence in current climate models. *Geophys. research letters* **46**, 1861–1869 (2019).
